# Supplementary material for: MetaRibo-Seq measures translation in microbiomes
Source: Nat Commun. 2020 Jun 29;11:3268. doi: 10.1038/s41467-020-17081-z (PMC7324362; doi:10.1038/s41467-020-17081-z)
Supplement: Supplementary file 10 — Supplementary Data 7 [file 41467_2020_17081_MOESM10_ESM.zip › File2/Confidence_VeryHigh_Taxonomy/1382_out.krona.html]

Javascript must be enabled to view this page.

members
magnitude
magnitudeUnassigned
count
unassigned
taxon
rank

1382\_out

8

superkingdom
2759
2

4751
kingdom
2

phylum
4761
2

2
class
451435

34478
order
2

2
34479
family

2
genus
4815

2
species

SRS143598\_contig\_number\_8743SRS149879\_contig\_number\_19089
109760

superkingdom
2
6

1239
phylum
6

186801
class
6

6
order
186802

family
186803
6

genus
572511
6

2

SRS012273\_contig\_number\_32373SRS013476\_contig\_number\_contig-100\_10718.10718
species
33038

4
1226324
species

SRS013215\_contig\_number\_contig-100\_3949.3949SRS015217\_contig\_number\_contig-100\_26709.26709SRS022137\_contig\_number\_18088SRS024549\_contig\_number\_1156
